# Parent and adolescent reports on emotional and peer problems in psychiatric outpatient setting using SDQ

**DOI:** 10.1192/j.eurpsy.2021.616

**Published:** 2021-08-13

**Authors:** A. Kocane, N. Bezborodovs

**Affiliations:** 1 Psychiatry, LLC Hospital Gintermuiza, Jelgava, Latvia; 2 Faculty Of Residency, Riga Stradins University, Riga, Latvia; 3 Department Of Psychiatry And Narcology, Riga Stradins University, Riga, Latvia; 4 Child Psychiatry Clinic, Children’s Clinical University hospital, Riga, Latvia

**Keywords:** adolescent, outpatient, SDQ, internalising

## Abstract

**Introduction:**

The Strengths and Difficulties Questionnaire (SDQ) is one of the most widely used screening instruments in child and adolescent psychiatry. Studies have shown that the parent is a better informant than the adolescent, both for externalising and internalising disorders (Goodman et al, 1997, 2000).

**Objectives:**

Aim of this study was to examine the prevalence of parent and adolescent reported internalising problems in outpatient child and adolescent psychiatry setting using SDQ and examine the differences between parent and adolescent reports.

**Methods:**

The study group was 101 adolescents (11-17 y.o.) and their parents, in 2 outpatient psychiatric care centres in Latvia. Internalising problems were assessed using SDQ parent and self-report version. When analyzing the score, 3rd and 4th band were defined as “high”.

**Results:**

The mean age of adolescent population was 14,04 years (SD 1,96) and N=54 were female. 60,4% of parents and 52,5% of adolescents reported high level of peer problems, 63,4% of parents and 51,5% of adolescents reported high level of emotional problems. Parent and adolescent report results were concordant in two thirds of cases. Adolescents reported high emotional and peer problems in 9% and 14% of cases respectively, when their parents did not. And on the contrary - 22% of parents reported high level of internalising problems when the adolescent did not.

**Conclusions:**

More than half of reports showed high levels of internalising problems. Every fifth parent reported a higher level of internalising problems than their adolescent. This agrees with previous findings that single informant (parent) reports might be more informative than multi-informant reports.

